# Foreign Correspondence

**Published:** 1848-06

**Authors:** 


FOREIGN CORRESPONDENCE.

Dr. David W. Yandell, so long our foreign correspondent, arrived at New York on the 22d of May, in the steamship Hermann, from Southampton, and is daily looked for in Louisville. He is bringing home with him copious notes on medical matters and men in Europe, and we hope will continue to favor us with his valued contributions. Mr. David C. Tandy, a most promising young Kentuckian, who has been pursuing his medical studies for several years in Europe, will succeed him as foreign correspondent of our Journal. Dr. Yandell returns to pursue his profession in Louisville.

